# Microsurgical vs. Endoscopic Excision of Colloid Cysts: An Analysis of Complications and Costs Using a Longitudinal Administrative Database

**DOI:** 10.3389/fneur.2017.00259

**Published:** 2017-06-09

**Authors:** Ian David Connolly, Eli Johnson, Layton Lamsam, Anand Veeravagu, John Ratliff, Gordon Li

**Affiliations:** ^1^Department of Neurosurgery, Stanford University School of Medicine, Palo Alto, CA, United States

**Keywords:** MarketScan, colloid cyst, endoscopy, microsurgery, adverse events, seizures

## Abstract

**Objective:**

Open microsurgical and endoscopic approaches are the two main surgical options for excision of colloid cysts. Controversy remains as to which is superior. Previous studies consist of small cohort sizes. This topic has not been investigated using national administrative claims data which benefits from larger patient numbers.

**Methods:**

Current Procedural Terminology (CPT) and International Classification of Disease version 9 (ICD-9) coding at inpatient visit was used to select for index surgical procedures corresponding to microsurgical or endoscopic excision of colloid cysts. Comorbidities, costs, and complications were collected.

**Results:**

We identified a total of 483 patients. In all, 240 were from the microsurgical cohort and 243 were from the endoscopic cohort. The two groups displayed similar demographic and comorbidity profiles. Thirty-day post-operative complications were also similar between groups with the exception of seizures and thirty-day readmissions, both higher in the open surgical cohort. The seizure rates were 14.7 and 5.4% in the microsurgical and endoscopic cohorts, respectively (*p* = 0.0011). The thirty-day readmission rates were 17.3 and 9.6% in the microsurgical and endoscopic cohorts, respectively (*p* = 0.0149). Index admission costs and 90-day post discharge payments were higher in patients receiving microsurgical excision.

**Conclusion:**

An analysis of administrative claims data revealed few differences in surgical complications following colloid cyst excision *via* microsurgical and endoscopic approaches. Post-operative seizures and thirty-day readmissions were seen at higher frequency in patients who underwent microsurgical resection. Despite similar complication profiles, patients undergoing microsurgical excision experienced higher index admission costs and 90-day aggregated costs suggesting that complications may have been more severe in this group.

## Introduction

Colloid cysts are benign tumors that most commonly appear in the third ventricle and account for 0.5–2.0% of all central nervous system tumors (1–5). They are composed of an outer fibrous layer and an inner epithelium of ciliated or mucin-producing cells (6, 7). In patients presenting with symptoms the mortality is estimated to be 3.1–12% (8). Overall mortality rate, regardless of presenting symptoms, is reported to be 1.2% (6, 8). Nearly 60% of these mucinous cysts are found incidentally during routine work-up for other neurological diseases and are asymptomatic (7, 8). In symptomatic cases, obstructive hydrocephalus may occur due to proximity with the foramen of Monro. Additional symptoms include coma, headaches, nausea, and vomiting (4, 9–11). Patient age, headache as a primary symptom, and hydrocephalus are significantly associated with symptomatic colloid cysts (12). More than half of the patients presenting with symptoms will need surgical intervention (12). Surgical treatment is indicated for cysts that are symptomatic, large, or associated with hydrocephalus. Close observation is considered for small asymptomatic lesions (13).

Typical surgical interventions include cerebrospinal fluid (CSF) shunting, cyst aspiration, microsurgical resection, and endoscopic resection (4, 14, 15). Although CSF shunting and cyst aspiration can transiently manage symptoms, complete removal through microsurgical or endoscopic resection is preferred to minimize cyst recurrence (14, 16). Complications from all types of surgical management include seizures, hematoma, infection, venous infarct, memory deficit, mutism, and hemiplegia (17). No consensus on which type of surgical resection is most effective has been reached. A review and meta-analysis by Sheikh et al. found that microsurgical resection results in a significantly higher rate of complete resection, decreased rate of recurrence, fewer reoperations, but higher overall rate of morbidity than endoscopic surgery (18). Microsurgical resection, particularly the transcortical approach, is additionally associated with increased risk of seizures (19). The endoscopic approach reduces the risk of infection, operation time, and hospital stay (20, 21).

Although studies have attempted to characterize patient demographics, comorbidities, and outcomes for microsurgical vs. endoscopic resection of colloid cysts, there is a gap in our current understanding. To our knowledge, the present study is the first to look at comorbidities and outcomes utilizing a national administrative database and the largest single report of microsurgical vs. endoscopic colloid cyst outcomes.

This knowledge is necessary to effectively guide best clinical practice and patient counseling.

## Materials and Methods

### Study Design and Data Source

We conducted a retrospective analysis of outcomes of for patients undergoing colloid cyst resection from the Thomson Reuters MarketScan Commercial Claims and Encounters and Medicare Supplemental databases, administered by Truven Health Analytics. The MarketScan dataset contains data from over 100 payers and includes inpatient, outpatient, and pharmacy services from a range of large employers, health plans, governmental organizations, and public organizations. All visits from multiple providers, including inpatient and outpatient services, are reconciled and recorded longitudinally for each patient. For this study, we utilized the inpatient and outpatient data tables from Marketscan years 2007 to 2015. The inpatient table includes encounters associated with an inpatient admission. It contains up to fifteen diagnosis codes (ICD-9) and up to fifteen procedure codes (primarily CPT). The outpatient table includes services rendered during a patient visit to a physician’s office, hospital outpatient facility, emergency room or other outpatient facility. It contains up to two diagnosis codes (ICD-9) and one procedure code (CPT).

Our group’s studies involving the MarketScan database were reviewed by the Stanford Institutional Review Board. Because the data source did not contain identifiable patient information, these studies were granted an exemption from the Institutional Review Board approval process.

### Setting and Participants

Between 2007 and 2015, we identified 483 patients from inpatient admission records containing a diagnosis of a colloid cyst (ICD-9 diagnosis code 742.2) with concurrent CPT codes for endoscopic excision (62162) or microsurgical excision (61516). If a patient had multiple procedures meeting the criteria, the first was considered the index procedure and the subsequent was considered a reoperation. These codes are included in Table 1.

**Table 1:** International Classification of Disease version 9 (ICD-9) and Current Procedural Terminology (CPT) codes used to identify cohort, comorbidities, and complications.

CPT and ICD-9 codes used to select cohort, comorbidities, and complications	
Other specified congenital anomalies of brain (ICD-9)	742.2
Supratentorial bone flap craniotomy for excision of cyst (CPT)	61516
Intracranial neuroendoscopy with excision of colloid cyst (CPT)	62162
Deep vein thrombosis (ICD-9)	451, 453.4, 453.8, 453.2, 453.1, 453.9
Dysphagia (ICD-9)	787.2
Dysrhythmia (ICD-9)	427, 426.1, 426, 426.3, 426.4, 426.5, 426.6, 426.7, 426.8
General neurological complication (ICD-9)	430, 431, 432, 433, 434, 435, 436, 438.2, 438.3, 438.4, 438.5
SAH (ICD-9)	430
Intracerebral hemorrhage (ICD-9)	431
Other and unspecified intracranial hemorrhage (ICD-9)	432
Occlusion and stenosis of precerebral arteries (ICD-9)	433
Occlusion of cerebral arteries (ICD-9)	434
Transient cerebral ischemia (ICD-9)	435
Acute, but ill-defined, cerebrovascular disease (ICD-9)	436
Hemiplegia/hemiparesis (ICD-9)	438.2
Monoplegia of upper limb (ICD-9)	438.3
Monoplegia of lower limb (ICD-9)	438.4
Other paralytic syndrome (ICD-9)	438.5
General neurosurgical complication (ICD-9)	997
MI (ICD-9)	410, 412, 998.0, 997.1, 411, 429.7
Osteoporosis (ICD-9)	733, V17.81, 731.3, V82.81
Obesity (ICD-9)	278
Other wound complication (ICD-9)	998.3, 998.81, 998.83, 998.4
Post-op infection (CPT)	10060, 10140, 10180, 12020, 12021, 20005, 21501, 22010, 22015
Post-op infection (ICD-9)	998.0, 998.5, 999.3
Pulmonary complication (ICD-9)	997.3, 518.4, 518.5, 518.7, 518.81, 518.82, 518.83, 518.84, 518.89, 519.1
Pulmonary embolism (ICD-9)	415.1
Seizure (ICD-9)	345, 780.3
Thromboemboli (ICD-9)	453.0, 453.4, 453.8,453.2, 453.3
Tobacco use (ICD-9)	305.1, V15.82, 989.84, 649.0
Wound dehiscence (ICD-9)	998.3
Wound hematoma (ICD-9)	998.1
Memory loss (ICD-9)	780.93
Meningitis (ICD-9)	320, 321, 322
Ventricular shunt (CPT)	62223

### Variables and Outcomes

Patient comorbidities were identified from outpatient records prior to index admission with appropriate ICD-9 diagnosis codes (Table 1). Additional outcomes included home-discharge rate, length of stay, complication rates, and related hospital payments, physician payments, and total payments from the inpatient records. Complications were collected from inpatient and outpatient records within 30 days after index admission using appropriate ICD-9 and CPT codes (Table 1). If a patient had any history of the complication, it was excluded from the analysis. In order to capture the long-term financial effects of adverse events, 90-day associated payments were aggregated from inpatient readmissions (total payments) and outpatient records within 90 days of index admission discharge.

### Statistical Analysis

Data preparation and analysis were performed using SAS software (version 9.3; SAS Institute, Inc., Cary, NC, USA). Tests of significance were performed using a Fisher’s exact test for categorical variables and a Student’s *t*-test for continuous variables with an alpha of 0.05. A Wilcox Rank-Sum test was used to analyze differences in costs. Odds ratios were calculated with the microsurgical group serving as the reference. For analysis of predictor variables of seizures and thirty-day readmissions, we performed a stepwise logistic regression model using a *p*-value cutoff of 0.05 adjusting for tobacco use, age ≥70, and sex.

## Results

### Patient Demographics

Between 2007 and 2015, an identified 483 patients received colloid cyst excision in the MarketScan database. Of these cases, 240 were performed *via* the microsurgical approach and 243 were performed endoscopically (Table 2). The mean age of the microsurgical cohort was younger than the endoscopic cohort though this difference was non-significant (38 vs. 41 years; *p* = 0.1172). Length of stay was slightly longer in the microsurgical group but was also non-significant (6.5 vs. 5.7 days; *p* = 0.2797). Interestingly, the percentage of patients discharged home was nearly identical in both groups. The comorbidity profile of both groups was also similar with no statistically significant differences (Table 3).

**Table 2:** Patient demographics.

Demographics	Microsurgical (*N* = 240)	Endoscopic (*N* = 243)	OR (CI)	*p*-Value
Age, mean (SD)	38 (18.4)	40.6 (17.9)	–	0.1172
Follow-up, mean (SD)	862.2 (742.3)	882.6 (794.6)	–	0.7716
Length of stay (nights), mean (SD)	6.5 (7.5)	5.7 (7.1)	–	0.2797
Discharge home, *N* (%)	206 (85.8)	213 (87.7)	1.17 (0.69–1.98)	0.5928
Male, *N* (%)	127 (55)	122 (54)	1.06 (0.73–1.54)	0.778

**Table 3:** Patient comorbidities.

Comorbidities	Microsurgical, *N* (%)	Endoscopic, *N* (%)	OR (CI)	*p*-Value
Tobacco use	30 (13)	19 (7.9)	0.58 (0.31–1.06)	0.0960
Osteoporosis	20 (8.7)	15 (6.3)	0.7 (0.35–1.41)	0.3806
Hypertension (Elixhauser)	73 (31.6)	72 (30)	0.93 (0.63–1.37)	1
CHF (Charlson)	5 (2.2)	4 (1.7)	0.77 (0.2–2.89)	1
COPD (Elixhauser)	42 (18.2)	41 (17.1)	0.93 (0.58–1.49)	0.8092
MI (Charlson)	6 (2.6)	5 (2.1)	0.8 (0.24–2.65)	1
Diabetes (Elixhauser)	21 (9.1)	18 (7.5)	0.81 (0.42–1.56)	1
Obesity	14 (6.1)	11 (4.6)	0.74 (0.33–1.68)	0.5404

### Adverse Events

Post-operative complications occurring within thirty days of index admission are shown in Table 4. Patients undergoing a microsurgical excision of a colloid cyst experienced a slightly higher overall complication rate compared to patients receiving endoscopic excision (48.6 vs. 44.3%). This difference was not statistically significant. The post-operative complications were similar between both groups of patients. The most common adverse events among both groups were general neurological complications (27%), seizures (10%), dysrythmia (9%), intracranial hemorrhage NOS (8%), pulmonary complications (7%), and general neurosurgical complications (6%). Between groups, the only statistically significant differences in post-operative outcomes were seizure occurrence and thirty-day readmission, both of which were lower in the endoscopic excision group. Seizures were experienced at a rate of 5.4% in the endoscopic group and 14.7% in the microsurgical excision group (*p* = 0.0011). Approximately 40% of post-operative seizures fell within the index inpatient admission. All cause thirty-day readmission was observed in 9.6% of endoscopic patients and 17.3% of microsurgical excision patients (*p* = 0.0149). Two patients in the microsurgical cohort (0.83%) and one in the endoscopic cohort (0.41%) underwent reoperation. Post-operative ventricular shunting rates were approximately the same in both groups with 3.5 and 2.5% in microsurgical and endoscopic groups, respectively. Overall, there were three index admission inpatient mortalities. Two occurred in a patient undergoing microsurgical excision (0.83%) and another occurred in a patient undergoing endoscopic excision (0.41%).

**Table 4:** Patient complications.

Complications	Microsurgical, *N* (%)	Endoscopic, *N* (%)	OR (CI)	*p*-Value
Wound infection	7 (3)	5 (2.1)	0.68 (0.21–2.18)	0.5697
Wound dehiscence	5 (2.2)	3 (1.3)	0.57 (0.14–2.42)	0.4966
Wound hematoma	10 (4.3)	9 (3.8)	0.86 (0.34–2.16)	0.8174
Other wound complication	5 (2.2)	4 (1.7)	0.77 (0.2–2.89)	0.7473
Delirium	3 (1.3)	2 (0.8)	0.64 (0.11–3.86)	0.6804
Pulmonary embolism	2 (0.9)	4 (1.7)	1.94 (0.35–10.7)	0.6858
DVT	6 (2.6)	7 (2.9)	1.13 (0.37–3.4)	1
Any thromboembolism	4 (1.7)	5 (2.1)	1.21 (0.32–4.55)	1
Pulmonary complication	18 (7.8)	17 (7.1)	0.9 (0.45–1.8)	0.861
General neurological complication	68 (29.4)	61 (25.4)	0.82 (0.54–1.23)	0.3531
Subarachnoid hemorrhage	5 (2.2)	2 (0.8)	0.38 (0.07–1.98)	0.277
Intracranial hemorrhage NOS	22 (9.5)	15 (6.3)	0.63 (0.32–1.25)	0.2307
Precerebral arterial occlusion	1 (0.4)	0 (0)	–	0.4904
Cerebral artery occlusion	9 (3.9)	9 (3.8)	0.96 (0.37–2.47)	1
Transient ischemia attack	0 (0)	1 (0.4)	–	1
Acute complication NOS	3 (1.3)	5 (2.1)	1.62 (0.38–6.84)	0.7246
Hemiplegia/hemiparalysis	2 (0.9)	2 (0.8)	0.96 (0.13–6.89)	1
General neurosurgical complication	18 (7.8)	12 (5)	0.62 (0.29–1.32)	0.2587
Iatrogenic stroke	6 (2.6)	9 (3.8)	1.46 (0.51–4.17)	0.6023
Post-op dysrhythmia	21 (9.1)	23 (9.6)	1.06 (0.57–1.97)	1
Post-op MI	6 (2.6)	4 (1.7)	0.64 (0.18–2.28)	0.5375
Post-op dysphagia	2 (0.9)	1 (0.4)	0.48 (0.04–5.32)	0.6171
Post-op seizure	34 (14.7)	13 (5.4)	0.33 (0.17–0.65)	0.0011
Post-op meningitis	10 (4.3)	12 (5)	1.16 (0.49–2.75)	0.8285
Post-op memory loss	11 (4.8)	12 (5)	1.05 (0.46–2.44)	1
Post-op ventricular shunting	8 (3.5)	6 (2.5)	0.71 (0.24–2.09)	0.5957
30-day readmission	40 (17.3)	23 (9.6)	0.51 (0.29–0.88)	0.0149
Any complication	107 (48.6)	99 (44.3)	0.84 (0.58–1.23)	0.3921

A stepwise logistic regression model revealed a variety of post-operative complications as risk factors for thirty-day readmission (Table 5). Post-operative meningitis and any embolism had the highest odds ratios (8.91 and 7.76, respectively). Post-operative seizures were also a large predictor of thirty-day readmission with an odds ratio of 6.38. Surgical procedure type did not enter into the model because it did not reach a statistically significant value. For post-operative seizures, endoscopic approach type had a statistically significant odds ratio of 0.32 after adjusting for age, sex, and tobacco use. This was similar to the crude value of 0.33 shown in Table 4.

**Table 5:** Stepwise logistic regression for predictors of 30-day readmission (adjusted for tobacco use, age ≥70, and sex).

Effect	Odds ratio (CI)
Any wound complication	5.66 (2.14–14.95)
Any embolism	7.76 (2.22–27.07)
Pulmonary complication	3.35 (1.15–9.60)
Neurosurgical complication	5.28 (1.93–14.46)
Post-op seizure	6.38 (2.73–14.91)
Post-op memory loss	5.65 (1.96–16.23)
Post-op meningitis	8.91 (2.77–28.71)

### Resource Utilization

No statistically significant differences in costs were seen among patients who experienced no complications (Table 6). Total index admissions costs among these patients were approximately $32,000 for both surgical approaches, and 90-day aggregated costs were approximately $1,700 and $2,000 in the microsurgical and endoscopic groups, respectively.

**Table 6:** Costs for patients experiencing one or more complication show increased costs in the microsurgical cohort.

	Payment type	Microsurgical, median (IQR)	Endoscopic, median (IQR)	Difference (%)	*p*-Value
No complications	Hospital payments	24,570 (19,826)	25,218 (17,452)	−648 (−2.57%)	0.6984
	Physician payments	3,603 (2,787)	3,862 (3,816)	−259 (−6.71%)	0.2568
	Total payments	31,780 (20,016)	31,791 (20,806)	−11 (−0.03%)	0.7708
	90 day post-discharge payments	1,661 (2,969)	2,027 (3,374)	−366 (−18.06%)	0.3328

≥1 complication	Hospital payments	38,846 (34,391)	31,404 (24,361)	7,441.83 (+23.7%)	0.018
	Physician payments	4,157 (3,530)	3,659 (3,193)	498.3 (+13.62%)	0.0487
	Total payments	46,911 (41,854)	39,083 (25,278)	7,828.06 (+20.03%)	0.0108
	90-day post-discharge payments	7,179 (29,773)	3,238 (13,150)	3,941.32 (+121.73%)	0.0082

When we examined the relationship between surgical approach and costs among patients who experienced at least one complication, we found that the microsurgical cohort experienced increased costs across all metrics (hospital, physician, total, and 90-day payments) that were statistically significant (Table 6). The most notable increase was in the 90-day post discharge payments which were approximately double that of the endoscopic group. Interquartile range tended to be larger in the microsurgical excision cohort.

## Discussion

The optimal surgical approach for excision of colloid cysts remains a topic of debate. There have been numerous reports on the outcomes of microsurgical and endoscopic approaches. Due to its rarity, studies on operative approaches in colloid cysts have been limited in both number and cohort size. The recent meta-analysis by Sheikh and colleagues comparing microsurgical and endoscopic approaches included 16 and 26 microsurgical and endoscopic studies, respectively (18). Altogether, the mean number of patients of these studies was approximately 30. To our knowledge, our study is the largest single study and the first to utilize an administrative database to study operative approach for colloid cysts.

Many previous studies support that the microsurgical approach maximizes the extent of surgical resection while the endoscopic approach is associated with shorter stays and lower complications (19, 22–25). However, many papers reporting endoscopic outcomes are from early studies when neuroendoscopy was still a new technique (26, 27). It thought that neuroendoscopic outcomes have since improved and that complete excision rates between microsurgical and endoscopic approaches are now more similar (28–31). Interestingly, we observed statistically significantly higher costs for the microsurgical cohort among patients experiencing complications despite similar overall complication profiles between the two approaches (Table 6). This suggests that patients undergoing microsurgical excision may have experienced more severe complications overall.

Sheikh et al. reported a variety of complications (seizures, memory deficits, venous infarction, subdural hematoma, intracerebral hematoma, arterial infarction, hemiparesis, and meningitis) (18). The largest difference in complication occurrence was seen with seizures in the endoscopic cohort (0.3% of patients) and the transcortical cohort (10.4% of patients). Interestingly, we also found a statistically significant difference in post-operative seizures with 5.4 and 14.7% of patients experiencing a seizure in the endoscopic and microsurgical cohorts, respectively (*p* = 0.0011). It is thought that the reduced risk seen in the endoscopic approach is due to the smaller cortical incision. For this reason, it is also believed that the transcallosal approach results in reduced rates of post-operative seizures since it uses natural tissue planes to reach the cyst (3, 32). However, this finding is not universal. A study by Milligan et al. described seizure rates of 8 and 25% for the transcortical and transcallosal approaches, respectively (33). Unfortunately, because there is no code specific to the transcortical or transcallosal approach, we were unable to shed light on this in our analysis. A previous study by our group on surgical outcomes following resection of meningioma, also using the MarketScan database, found a post-operative seizure rate of approximately 14% (34). Although a notable subset of patients develop post-operative seizures, it is important to note the strong evidence against the efficacy of prophylactic anti-epileptic drugs (35). Our logistic regression analysis revealed that post-operative seizures significantly increases the odds of thirty-day readmission. This result may explain the difference observed in thirty-day readmissions between the two surgical cohorts as seizures were nearly three times as frequent in the microsurgical group.

Despite small differences seen among other complications in Sheikh et al., our analysis was unable to capture these likely due to limitations in sample size and the sensitivity of diagnosis coding. We were also unable to find a major difference in mortality, reoperation, and post-operative shunt dependence. Our low rates of reoperation may be due to our lower follow-up which averaged approximately 870 days or 2.4 years. The average follow-up time in Sheikh et al. was about 4 and 3 years in the microsurgical and endoscopic surgical cohorts, respectively. Several of our other results were within range of what was reported in Sheikh et al. Our rate of post-operative memory loss was approximately 5% for both surgical groups. This was similar to what was reported by Sheikh and colleagues (4.9–5.7%). The rate of meningitis in our endoscopic group was slightly higher than what was reported in the latter study (5 vs. 2.7%). The rate of hemiplegia was less than 1% in our cohorts, which was similar to the range reported in Sheikh et al. (0.9–1.9%).

### Limitations

There are several limitations of our study that are inherent to administrative databases. First, as stated previously, costs differences were observed despite similar complication profiles for the two surgical approaches. This discrepancy highlights our inability to precisely determine disease severity from ICD-9 diagnosis codes alone, a common limitation of administrative data.

Second, the inherent selection bias of the database presents a significant limitation. Because MarketScan is a private insurance database, uninsured patients are not included. Thus, long-term outcomes may not be captured if a patient drops insurance coverage. Patients covered by Medicaid are similarly not included since most do not have additional private coverage. Because of the retrospective nature of the study, it is also possible that a particular surgical approach was more favored for tumors and/or centers with certain characteristics (i.e., large vs. small tumor size, academic vs. non-academic centers). Thus, our cohorts may not be reflective of the true population.

Third, the specificity of our analysis was limited to the granularity of the coded diagnoses, procedures, and the variables that were available in the database. For this reason, we were unable to investigate approach subtypes (i.e., transcortical, transcallosal) as was done in Sheikh et al. Many variables such as, patient ethnicity, cyst size, operative time, and extent of resection are not available in the database. Mortality is available but is only captured in the inpatient setting. Thus, we were unable to control for the aforementioned variables.

## Conclusion

To our knowledge, this study is the first to investigate post-operative outcomes and costs of microsurgical and endoscopic colloid cyst excision using an administrative database. Despite previous reports suggesting that endoscopic approaches are associated with lower complication rates, we were unable to find major differences with the exceptions of post-operative seizures and 30-day readmission. Both of these outcomes were higher in the microsurgical cohort. We did observe increased costs in patients undergoing the microsurgical approach. This suggests that this group may have experienced more severe complications than the endoscopic cohort despite having similar rates of ICD-9-coded complications. Administrative databases appear to have limited sensitivity to detect subtle differences in procedural outcomes. Care should be taken to evaluate the overall risks and benefits of both approach types for each patient.

## Author Contributions

Study conception and design: JR, GL, and AV. Analysis of data; manuscript preparation: IC, EJ, and LL. Manuscript revision and feedback: JR, GL, and AV. Final approval of submitted work; agreement to be accountable for published work: IC, EJ, LL, JR, GL, and AV.

## Conflict of Interest Statement

The authors declare that the research was conducted in the absence of any commercial or financial relationships that could be construed as a potential conflict of interest. The reviewer, TH, and handling editor declared their shared affiliation, and the handling editor states that the process nevertheless met the standards of a fair and objective review.

## Abbreviations

CPT, Current Procedural Terminology; CSF, cerebrospinal fluid; ICD-9, International Classification of Disease version 9.
